# The progress of research on vacancies in HMF electrooxidation

**DOI:** 10.3389/fchem.2024.1416329

**Published:** 2024-06-14

**Authors:** Zhikai Chen, Gan Zhang, Jinxia Jiang, Xin Feng, Wei Li, Xiaohong Xiang, Gan Linling

**Affiliations:** ^1^ School of Chemistry and Chemical Engineering, Chongqing University, Chongqing, China; ^2^ Chongqing Medical and Pharmaceutical College, Chongqing, China; ^3^ The Second Affiliated Hospital of Chengdu Medical College, China National Nuclear Corporation 416 Hospital, Chengdu, China; ^4^ Institute of Fundamental and Frontier Sciences, University of Electronic Science and Technology of China, Chengdu, China; ^5^ School of Materials and Energy, University of Electronic Science and Technology of China, Chengdu, China

**Keywords:** defect engineering, 5-hydroxymethylfurfural, electrocatalytic reaction, anionic vacancies, cationic vacancies

## Abstract

5-Hydroxymethylfurfural (HMF), serving as a versatile platform compound bridging biomass resource and the fine chemicals industry, holds significant importance in biomass conversion processes. The electrooxidation of HMF plays a crucial role in yielding the valuable product (2,5-furandicarboxylic acid), which finds important applications in antimicrobial agents, pharmaceutical intermediates, polyester synthesis, and so on. Defect engineering stands as one of the most effective strategies for precisely synthesizing electrocatalytic materials, which could tune the electronic structure and coordination environment, and further altering the adsorption energy of HMF intermediate species, consequently increasing the kinetics of HMF electrooxidation. Thereinto, the most routine and effective defect are the anionic vacancies and cationic vacancies. In this concise review, the catalytic reaction mechanism for selective HMF oxidation is first elucidated, with a focus on the synthesis strategies involving both anionic and cationic vacancies. Recent advancements in various catalytic oxidation systems for HMF are summarized and synthesized from this perspective. Finally, the future research prospects for selective HMF oxidation are discussed.

## 1 Introduction

Amidst the escalating global demand for energy and mounting environmental concerns, there has been a rapid surge in the development of renewable green energy storage and conversion technologies (Murray et al. 2009). Electrochemical water splitting has emerged as a promising strategy for green hydrogen production (Suh et al., 2012; Xiao et al., 2021; Yan et al., 2023). However, the sluggish kinetics associated with the oxygen evolution reaction (OER) poses a significant challenge, severely limiting the energy utilization efficiency of water splitting devices. Regarding OER, under standard conditions (that is, a temperature of 25°C, a pressure of 1 atm, and a concentration of all reactants and products at 1 mol/L), its standard potential (referenced to the standard hydrogen electrode) is conventionally deemed to be 1.23 V. However, given that OER is a complex, multi-step, and multi-electron transfer process, its actual reaction potential is influenced by kinetic limitations and overpotential effects. Consequently, in practical applications, a higher voltage is necessary to achieve efficient OER(Sun et al., 2022; Wang et al., 2022; Ma et al., 2023). In response to these challenges, there has been a shift in focus towards biomass-derived compounds such as furfural, furfuryl alcohol, 5-hydroxymethylfurfural (HMF), and glucose, due to their promising thermodynamic properties (Lu et al., 2022; Wang et al., 2022; Liu et al., 2023; Xiang et al., 2024). These compounds not only offer potential alternatives to OER but also present opportunities to maximize current efficiency while yielding high-value-added products, thereby offering novel solutions to energy conversion and environmental remediation challenges. Among these biomass-derived compounds, HMF holds particular significance as a crucial link between fossil-based chemicals and biomass resources. Its catalytic oxidation product, 2,5-furandicarboxylic acid (FDCA), holds immense application potential due to its structural similarity to terephthalic acid (TPA) derived from petroleum (Zhou et al., 2021). This similarity makes it instrumental in synthesizing biodegradable plastics, thereby carrying substantial implications for sustainable materials preparation. The electrocatalytic oxidation of HMF (HMFOR) for FDCA production has emerged as a focal point of research due to its mild reaction conditions and the absence of strong oxidants (Li et al., 2022; Li et al., 2022). By coupling HMFOR with the hydrogen evolution reaction (HER), not only can energy consumption for hydrogen production be significantly reduced, but substantial progress can also be made in industrial production and scientific research (Zhou et al., 2021). Therefore, gaining deeper insights into the catalytic mechanisms of HMFOR will provide crucial guidance and support for the development of highly efficient electrocatalysts (Wu et al., 2021).

In 1897, the case of Tate v. Latham and Son marked the first legal definition of “defect” as the absence or lack of something essential to completeness (Li et al., 2020; Yan et al., 2022). In contemporary scientific research, defects hold pivotal significance in materials science and catalysis. Defects, referring to imperfections within crystal structures, manifest in various forms, including point defects, line defects, and planar defects. These imperfections exert profound impacts on the physical, chemical, and electronic properties of materials, thus igniting widespread interest in catalysis research. Surface defects, considered critical in electrocatalyst design, serve as key factors in modulating catalytic activity and selectivity (Ares and Novoselov 2022; Yan et al., 2022). Among them, point defects, which encompass vacancies, substitutions, insertions, and interstitial atoms within the crystal lattice, can influence the electronic structure and surface reactivity of catalysts via doping or inherent mechanisms (Xiang et al., 2019; Xiang and Wang 2022; Huang et al., 2023; Wang et al., 2023). Line defects, such as dislocations and grain boundaries, constitute one-dimensional imperfections altering the local electronic environment and surface morphology, potentially affecting catalytic activity. Similarly, planar defects, including lattice mismatches and stacking faults, represent two-dimensional deviations in crystal structures that may also play significant roles in catalyst activity (Jia et al., 2018; Yan et al., 2021; Lopez-Polin et al., 2022). Despite the potential importance of defects in catalyst design, their complexity presents challenges. The effects of defect type, quantity, and location on catalytic performance depend on various factors such as material composition, synthesis methods, and reaction conditions. The HMFOR process occurs at the surface/interface of the catalyst, where defects facilitate the transport and formation of reactants, intermediates, and products (Wei et al., 2022). Widely present in heterogeneous/amorphous nanomaterials, defects can adjust the electronic structure and coordination environment of electrocatalysts, thereby altering reactant adsorption energy and ultimately enhancing electrocatalytic performance. For instance, anionic and cationic vacancy defects provide promising avenues for fine-tuning the performance of electrocatalytic materials, particularly in catalyzing electrochemical reactions. By strategically controlling the presence and distribution of defects, researchers can precisely adjust the electronic structure and surface properties of electrocatalysts, thus enhancing their catalytic activity and selectivity. This targeted approach holds tremendous potential for improving the efficiency and sustainability of electrochemical processes (Li et al., 2022; Liu et al., 2022; Xiao et al., 2023).

In this concise review, we provide a brief overview of the oxidation mechanism of HMF, followed by a focused discussion on the preparation and characterization of materials possessing both anionic and cationic vacancies. Furthermore, we delve into the influence of anionic and cationic vacancies on the HMFOR process, elucidating their structure-activity relationships. Finally, we highlight the prospects and challenges of anionic and cationic vacancy defects, covering aspects such as controllable synthesis, *in situ* characterization, and practical applications.

## 2 Oxidation mechanism of HMF

**FIGURE 1:**
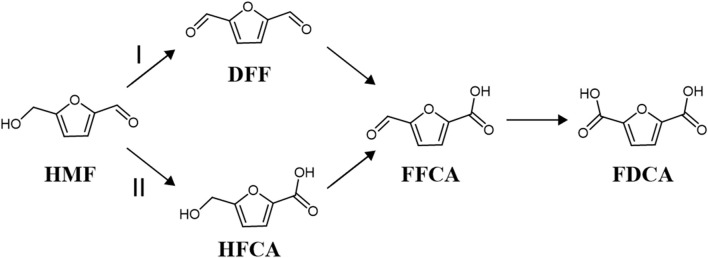
Pathway of HMF oxidation.

Due to the versatile chemical nature of HMF, attributed to the presence of both hydroxyl and aldehyde functional groups on its furan ring, it serves as a highly reactive precursor for various oxidation reactions, leading to the formation of a diverse range of high-value-added products and more stable derivatives (Guo et al., 2023). These include but are not limited to 2,5-diformylfuran (DFF), FDCA, and 5-hydroxymethyl-2-furancarboxylic acid (HFCA). Among these products, FDCA stands out as a particularly significant compound due to its broad applications in the synthesis of biodegradable plastics, pharmaceuticals, and other specialty chemicals (Jiang et al., 2016; Jiang et al., 2023). The oxidation of HMF to FDCA is a complex process that involves multiple reaction pathways and intermediates. One widely recognized pathway is the electrochemical oxidation of HMF (HMFOR), which occurs through the oxidation of both hydroxyl and aldehyde groups present in the HMF molecule. This electrochemical transformation, characterized by a six-electron transfer process, can proceed via two distinct routes, namely, Pathway I and Pathway II (Figure 1). In Pathway I, the oxidation of HMF initiates with the hydroxyl group, leading to the formation of DFF as the primary intermediate. This intermediate undergoes further oxidation to produce FFCA, which ultimately yields FDCA. On the other hand, Pathway II involves the initial oxidation of the aldehyde group of HMF, resulting in the formation of HMFCA as the primary intermediate. Similar to Pathway I, subsequent oxidation steps lead to the formation of FFCA and ultimately FDCA. The efficiency and selectivity of the conversion of HMF to FDCA are influenced by various factors, including the pH of the reaction medium, the applied voltage in electrochemical processes, and the catalytic properties of the catalyst (Gao et al., 2021). Understanding these factors and their impact on the oxidation mechanism of HMF is crucial for optimizing reaction conditions and developing efficient catalytic systems for FDCA production. Further research into the intricacies of these reaction pathways and the role of catalysts in modulating their kinetics holds significant promise for advancing the sustainable synthesis of FDCA from biomass-derived HMF (Yang and Mu 2021).

## 3 Anion vacancies promote HMFOR

The detection of vacancy formation relies primarily on Electron Paramagnetic Resonance (EPR) technique, also referred to as Electron Spin Resonance (ESR). This method is utilized for studying unpaired electrons within materials. In EPR, the sample is placed within a strong magnetic field and subjected to microwave radiation. The interaction between the spin of unpaired electrons and the applied magnetic field leads to resonance absorption. By measuring and analyzing the intensity and frequency of this resonance signal, one can deduce the presence of vacancies in the sample, along with their concentration and distribution. The generation of anionic vacancies in metal-based materials can arise through various mechanisms, each exerting a pivotal influence on material characteristics and behavior (Yang et al., 2023; Rezaei et al., 2024). Thermal activation represents a prevalent mechanism wherein elevated temperatures empower atoms within the metal lattice with adequate energy to surmount energy barriers, thus facilitating the migration of specific anions and leaving vacancies in their wake. This phenomenon is commonly observed during heat treatments or annealing processes, where the application of high temperatures fosters atomic mobility and the formation of lattice imperfections (Wu et al., 2021; Ma et al., 2024). Moreover, radiation-induced damage serves as another notable pathway for anionic vacancy formation. Exposure to radiation, such as neutrons or gamma rays, can instigate atomic displacement and structural modifications within the metal crystal lattice, thereby engendering anionic vacancies (Liu et al., 2019; Ma et al., 2024). This occurrence is frequently encountered in environments like nuclear reactors, radioactive material handling, or other nuclear applications. Furthermore, chemical reactions also contribute to the genesis of anionic vacancies. For instance, in certain oxidation-reduction reactions involving metals, interaction with oxygen may lead to the liberation of oxygen atoms from the lattice, consequently generating vacancies and ensuing anionic vacancies. In summary, the formation of anionic vacancies in metal-based materials is a multifaceted process influenced by factors such as high temperatures, radiation exposure, or chemical reactions, all of which induce atomic mobility or release, ultimately culminating in the creation of anionic vacancies (Xiong et al., 2024; Yang et al., 2024).

Anionic vacancies, predominantly oxygen vacancies (O_v_), play a pivotal role in shaping the physicochemical properties of metal-based materials. Leveraging the low formation energy of O_v_, defective metal oxides undergo significant modifications in their physical and chemical traits (Liu et al., 2022; Sha et al., 2023). This deliberate adjustment of catalysts’ crystal and electronic structures augments their adsorption capacity, thereby markedly enhancing their catalytic efficacy. Introducing O_v_ onto the surface of metal oxides can be accomplished through various techniques, including non-in-situ catalyst synthesis followed by high-temperature reduction, plasma treatment, laser irradiation, air-assisted thermal shock, and others (Yan et al., 2021). The efficacy of electrocatalysts hinges greatly on the success of these synthesis methodologies, which may sometimes prove intricate, time-intensive, and challenging to execute. Nano-casting methods offer distinct advantages in terms of simplicity, precision, and adaptability, facilitating the *in situ* generation of O_v_ during the fabrication of mesoporous metal oxides (Ye et al., 2022).

**FIGURE 2:**
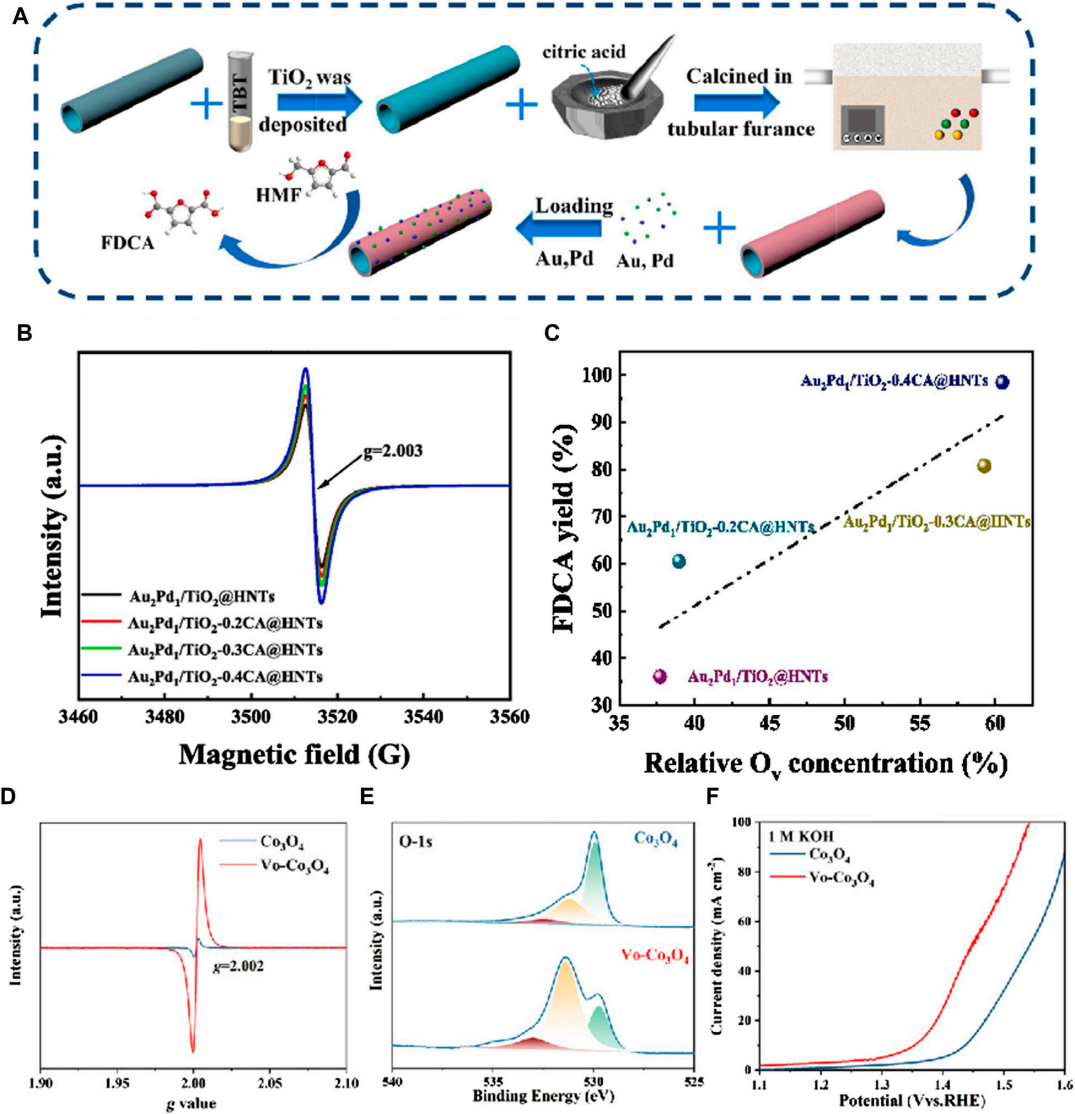
**(A)** The schematic representation of the catalyst preparation procedure; **(B)** EPR patterns of Au_2_Pd_1_/TiO_2_-xCA@HNTs catalysts; **(C)** Correlation analysis of O_v_ concentration and FDCA yield (Liu et al., 2023); **(D)** EPR spectra of Co_3_O_4_ and V_o_-Co_3_O_4_; **(E)** O 1s XPS spectra fitting for Co_3_O_4_ and V_o_-Co_3_O_4_; **(F)** LSV curves of V_o_-Co_3_O_4_ and Co_3_O_4_ in 1 M KOH with 50 mM HMF at a scan rate of 5 mV·s^-1^ (Liu et al., 2022).

Wu et al. (2024) reported the first synthesis of mesoporous Sc_2_O_3_ electrocatalysts rich in O_v_ via a nano-casting method, enabling the efficient and selective oxidation of HMF to FDCA. This innovation enabled the effective and selective oxidation of HMF to FDCA. The abundance of O_v_ within the mesoporous Sc_2_O_3_ influenced its electronic structure, thereby enhancing the adsorption and electrochemical oxidation of HMF. This was supported by both experimental observations and theoretical calculations. The outcomes revealed that in alkaline environments with 10 mM HMF, the mesoporous V_o_-Sc_2_O_3_ catalyst with a high surface area demonstrated remarkable efficiency and selectivity in converting HMF to FDCA, achieving optimal yields and Faraday efficiencies at 1.46 V (vs RHE). Liu et al. (2023) designed TiO_2_-coated halloysite nanotubes (HNTs) with different concentrations of Ov, synthesized using citric acid (CA) assistance, for the loading of AuPd nanoparticles (As Figure 2A). In Figure 2B, at g = 2.003, each spectrum exhibits a distinctive signal attributed to the F center (an electron trapped in O_v_), with the concentration of O_v_ increasing proportionally to the amount of CA added. As shown in Figure 2C, as the O_v_ concentration rises, so does the yield of FDCA. The adjustable O_v_ concentration effectively induces the synthesis of AuPd nanoparticles and TiO_2-x_CA@HNTs support in the catalyst, facilitating the oxidation of HMF to the bio-plastic monomer FDCA in water. This synthesis achieves a remarkable FDCA yield of 98.4%, an FDCA formation rate of 900.9 mmol·g^-1^·h^-1^, and a turnover frequency of 441.2 h^-1^. The presence of O_v_ not only increases the adsorption capacity of substrates and key intermediates, but also promotes the adsorption and activation of molecular oxygen, generating oxygen active superoxide radicals. Catalyst characterization and experimental studies revealed that the catalytic performance was significantly influenced by the O_v_ concentration. Lu et al. (2022) synthesized Co_3_O_4_ nanosheets via electrodeposition and introduced O_v_ via plasma treatment under an argon atmosphere. Analysis of the O_v_-Co_3_O_4_ samples revealed distinct EPR spectra with a characteristic g-value of 2.002, indicative of electrons trapped within O_v_ (Figure 2D). These vacancies originated from chemisorbed oxygen species on the surface, forming defect oxide groups. The O_v_ content was quantified via O^1s^ spectra at 531 eV, revealing a notable O_v_ concentration (78%) in the O_v_-Co_3_O_4_ nanosheets (Figure 2E). During the hydrogenation of HMF (HMFOR), O_v_ species adsorbed OH- ions from the electrolyte, facilitating their coupling with organic molecules. This interaction led to saturated Co-o coordination and an elevation in the Co oxidation state. Such direct coupling mechanisms effectively lowered the reaction barrier of the rate-determining step, thereby bolstering the electrocatalytic efficiency of HMFOR. Consequently, the O_v_-Co_3_O_4_ catalysts exhibited heightened activity and a reduced oxidation potential (1.37 V_RHE_) compared to pristine Co_3_O_4_ (1.44 V_RHE_) (Figure 2F). Wang et al. (2022) used laser ablation strategy to manufacture nickel oxide (O_v_-NiO) with different O_v_ contents. 100 mM HMF was oxidized 10 times at a constant voltage of 1.42V, and O_v_-NiO still maintained excellent catalytic activity. NiOOH was more easily reconstructed on the surface of O_v_-NiO. This is because under alkaline conditions, OH^−^ can fill oxygen vacancies and pre oxidize low valent Ni, forming NiOOH synergistic adsorption of HMF, resulting in higher stability of the material.

## 4 Cationic vacancies promote HMFOR

Cationic vacancies, characterized by the absence of metal ions within the crystalline lattice and commonly situated proximate to neighboring metal atoms, represent a fundamental aspect of solid-state physics and materials science. The genesis of these vacancies is underpinned by a multitude of intricate mechanisms (Ding et al., 2023; Xu et al., 2024; Zhou et al., 2024). Foremost among these mechanisms is thermal activation, a prevalent pathway wherein heightened temperatures catalyze the mobility of cations within the lattice, engendering the reconfiguration of adjacent cations and thus culminating in the creation of cationic vacancies. Additionally, chemical reactions serve as another significant avenue for the formation of cationic vacancies. Notably, in reduction reactions, the reduction of metal atoms liberates cations, thereby facilitating the generation of cationic vacancies (Zhang et al., 2020; Guo et al., 2024). The impact of radiation damage cannot be overstated in the context of cationic vacancy formation. Exposure to radiation induces perturbations within the crystal lattice, prompting the displacement or impairment of cations and thus fostering the emergence of cationic vacancies. Moreover, chemical adsorption emerges as a pertinent factor contributing to vacancy formation. Through chemical bonding with lattice cations, adsorbed molecules or atoms catalyze the rearrangement of neighboring cations, ultimately precipitating the formation of cationic vacancies (He et al., 2022; Kong et al., 2024). In synthesis, the formation of cationic vacancies is intricately intertwined with factors such as elevated temperature, chemical reactions, radiation exposure, and chemical adsorption, all of which modulate the dynamics of cation movement, damage, or removal within the crystal lattice, thereby orchestrating the creation of cationic vacancies (Wu et al., 2021).

**FIGURE 3:**
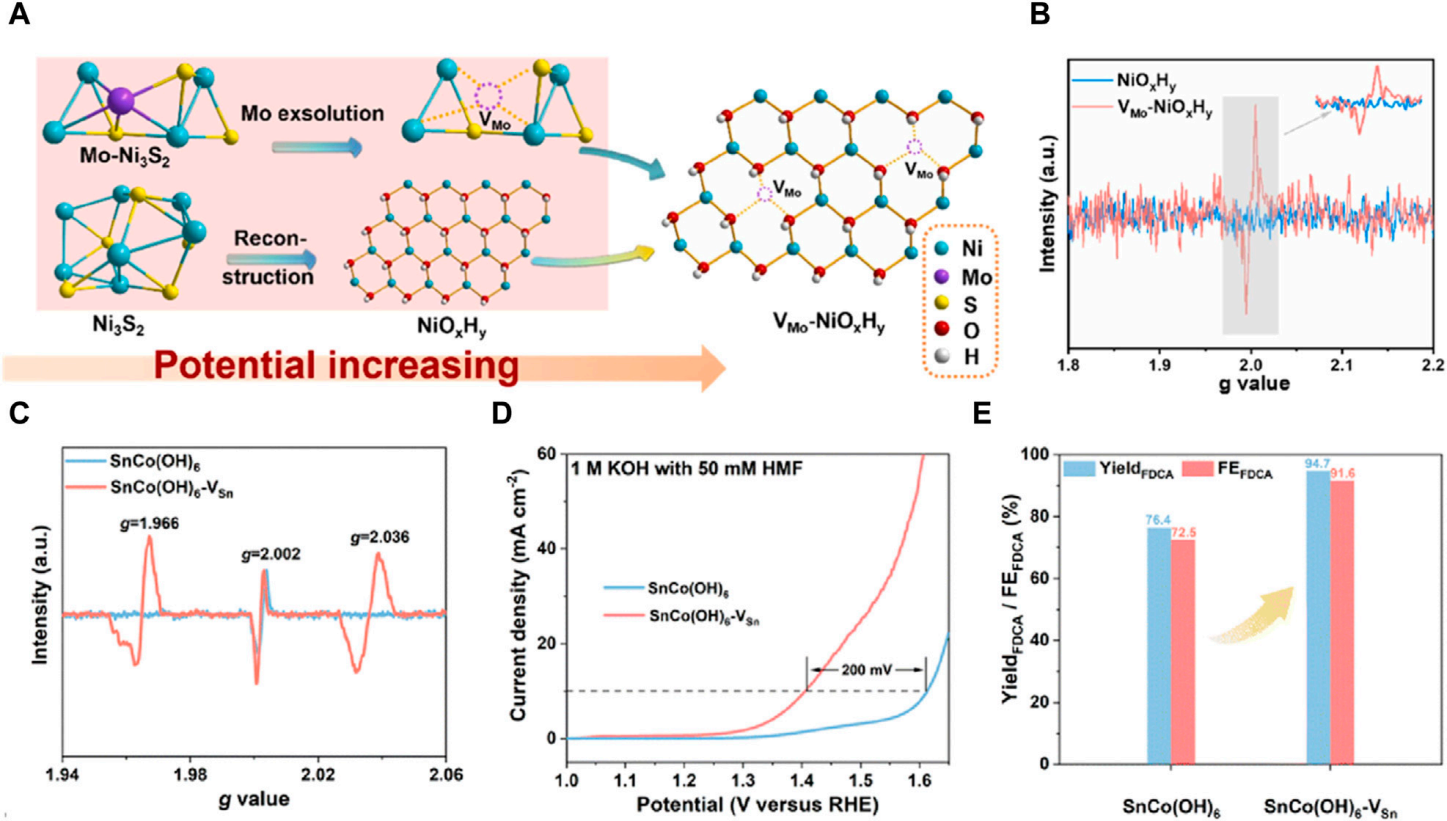
**(A)** The scheme of the relationship between electrocatalyst evolution and potential; **(B)** EPR spectra of V_Mo_-NiO_x_H_y_ and NiO_x_H_y_ (Jiang et al., 2024); **(C)** EPR spectra of SnCo(OH)_6_ and SnCo(OH)_6_-V_Sn_; **(D)** LSV curves for SnCo(OH)_6_ and SnCo(OH)_6_-V_Sn_ in 1 M KOH with 50 mM HMF; **(E)** yield and FE of FDCA at 1.47 V_RHE_ for SnCo(OH)_6_ and SnCo(OH)_6_-V_Sn_ (Peng et al., 2023).

Jiang et al. (2024) devised a novel method to generate cationic vacancies by *in situ* removing molybdenum from the pre-catalyst (Figure 3A). The EPR spectra of V_Mo_-NiO_x_H_y_ exhibited a strong electron paramagnetic resonance intensity at g = 2.003, whereas no VMo EPR signal was observed in the pristine NiOxHy (Figure 3B), confirming the formation of cationic V_Mo_ defects without the generation of sulfur vacancies during the reconstruction process. Compared to the pristine NiO_x_H_y_, the resulting vacancy-enriched V_Mo_-NiO_x_H_y_ catalyst demonstrated a significant threefold increase in current density, accompanied by a notable reduction in the onset voltage for water decomposition. This enhancement in performance can be attributed to several factors: vacancy manipulation induced the formation of Ni^3+^-O active species during the pre-oxidation process, optimized substrate adsorption, and modification of the nickel electronic coordination structure due to vacancy generation. During the electrochemical activation process, the *in situ* leaching of Mo and S triggers the formation of cation vacancy defects and subsequent surface reconstruction. This innovative approach exploits cation defects to enhance the creation of active sites, thereby optimizing the pre-oxidation process of highly efficient HMFOR catalysts. Peng et al. employed cobalt-based perovskite hydroxide as a model material and utilized a straightforward plasma etching strategy to introduce cationic vacancies in SnCo(OH)_6_. The lower chemical bonding energy and lattice energy of Sn(OH)_4_ made plasma treatment effective in liberating A-site Sn^4+^ ions from the perovskite structure. This led to the creation of amorphous surface structures rich in Sn cation vacancies. Due to structural dislocation, both samples exhibited a characteristic oxygen vacancy peak at g = 2.002. Following plasma etching, SnCo(OH)_6_-V_Sn_ displayed two additional vacancy peaks at g = 1.966 and g = 2.036, indicating the emergence of Sn cation vacancies (Figure 3C). The presence of these vacancies caused a 200 mV shift in the oxidation potential of HMFOR at the same current density (Figure 3D). Consequently, both the yield and Faraday efficiency were enhanced compared to the original configuration (Figure 3E) (Peng et al., 2023). Lu et al. (2020) enriched the abundance of defect-rich interface sites within three-dimensional layered nanostructured NiO-Co_3_O_4_ electrocatalysts and explored their catalytic efficacy for HMF electrooxidation. This interface-mediated enhancement resulted in an increased presence of cationic vacancies, which in turn modified the electronic properties of Co and Ni atoms, leading to an elevated oxidation state of Ni. The NiO-Co_3_O_4_ catalyst exhibited remarkable efficiency in catalyzing HMF oxidation, demonstrating a reduced onset potential of 1.28 VRHE. Moreover, the mechanisms underlying the electrocatalytic oxidation process were elucidated using *in situ* surface-enhanced vibrational spectroscopy via sum-frequency generation.

## 5 Conclusion and outlook

Exploiting anionic and cationic vacancy defects in the oxidation of HMF represents a promising avenue for enhancing reaction kinetics and catalytic performance. Engineered catalysts with customized defects undergo surface modifications that facilitate enhanced adsorption of reactants and intermediates, thereby expediting the conversion process. Moreover, the introduction of vacancies allows for precise tuning of the catalyst’s electronic structure, optimizing charge transfer dynamics and catalytic efficiency. This presents a significant opportunity for advancing more efficient and environmentally sustainable methods for HMF conversion, vital for producing valuable chemicals and fuels from biomass. However, realizing the practical application of defect-engineered catalysts for HMF oxidation poses several challenges. Achieving precise control over the spatial distribution and density of anionic and cationic vacancies within the catalyst matrix is paramount, necessitating a deep understanding of synthesis methodologies and mechanisms, along with rigorous optimization of experimental conditions. Additionally, ensuring the durability and long-term stability of defect-engineered catalysts in demanding reaction environments remains a significant obstacle, particularly for scaling up to industrial applications where sustained performance is crucial. Adding controlled amounts of impurity atoms or ions allows for the adjustment of both the electronic and lattice structures of materials, thereby promoting vacancy stability. Doping alters the density of electron clouds within the lattice, decreasing the energy needed for vacancy formation and subsequently reducing the material’s internal energy. Moreover, the widespread application of interface engineering techniques further reinforces vacancy structure stability. Through the design of surface modifications, coatings, or interfacial bonding layers, alterations in the material’s surface energy and reactivity occur, effectively curbing vacancy diffusion and aggregation, and ultimately enhancing vacancy structure stability.

In conclusion, while the potential advantages of utilizing anionic and cationic vacancy defects in HMF conversion are compelling, addressing challenges related to controlled synthesis, stability, and scalability is imperative. By overcoming these obstacles, researchers can unlock the full potential of defect-engineered catalysts and drive the development of efficient, sustainable technologies for converting biomass-derived feedstocks like HMF into high-value chemical products. Moreover, exploring the potential application of defect-engineered catalysts in other catalytic processes beyond HMF oxidation could further broaden their utility and impact in future industrial applications.
